# Posterior hemivertebra resection and reconstruction for the correction of old AO type B2.3 thoracic fracture kyphosis: A case report

**DOI:** 10.3389/fsurg.2022.945140

**Published:** 2022-08-11

**Authors:** Fanchao Meng, Xun Zhang, Tiantian Chen, Zhao Li, Yushi Fang, Wei Zhao, Jiaxing Xu

**Affiliations:** ^1^ Department of Orthopedics, The First Affiliated Hospital of Harbin Medical University, Harbin China; ^2^ Department of Orthopedics, Foresea Life Insurance Guangzhou General Hospital, Guangzhou, China

**Keywords:** hemivertebral, resection and reconstruction, thoracic fracture kyphosis, osteotomy, orthopedic

## Abstract

**Background:**

Post-traumatic malunion is one of the main causes of kyphosis and usually has serious consequences. We report a case of kyphosis caused by an old AO type B2.3 thoracic fracture, which was corrected with posterior hemivertebra resection and reconstruction.

**Case presentation:**

A 41-year-old male was diagnosed with kyphosis caused by an old AO type B2.3 thoracic fracture. Preoperative examination and preparation were performed. His exam images showed a comminuted fracture in the left half of the T12 vertebral body, while chance-type fractures were seen in the right half of T12 vertebral body and its accessories. During the operation, posterior hemivertebra resection and reconstruction techniques were used to remove nearly half of the left vertebral body of the affected vertebra, preserve the right vertebral body and the facet joints of the affected vertebra, correct the kyphosis, and rebuild spinal stability. The patient's low back pain was completely relieved, and his thoracic kyphosis was corrected at the seventh post-operative day. CT reconstruction of the spine showed that the residual vertebrae healed well during his nine- and 18-month follow-ups. Continuous callus formation was observed inside and outside of the titanium cage at the reconstructed site, and there was no sign of subsidence of the titanium cage. The heights between the vertebrae were restored to within normal ranges and the physiological curvature of the thoracolumbar spine was achieved. The patient recovered well.

**Conclusion:**

This operation preserved the hemivertebral body and facet joints, and maintains intervertebral height and local stability, thus avoiding titanium cage collapse, titanium cage movement, and other complications. This surgical approach is ideal for treating complex thoracic vertebral kyphosis caused by old fractures, and is worth utilizing in the clinic.

## Introduction

Kyphosis is commonly seen in cases of congenital vertebral malformation, old spinal tuberculosis, old spinal trauma, ankylosing spondylitis, Scheuermann's disease, and other diseases. Post-traumatic malunion is the most common cause of kyphosis (1). Delayed or incorrect treatment of spinal fractures can lead to localized kyphosis. The AO type B and C fractures are types that require surgical treatment. Load sharing classification (LSC) is used to describe the severity of spinal fractures. LSC scores the degree of vertebral comminution, displacement of fracture fragments, and correction angle of the kyphosis. Fractures with an LSC >6 require anterior column reconstruction surgery to prevent kyphosis (2). Traumatic kyphosis can be classified as a mild deformity or rigid deformity based on local healing. Mild deformities are due to bony nonunion within the vertebral body or disruption of intervertebral tissue adjacent to the affected vertebra, which leads to persistent local fretting. Combined anterior and posterior approaches are usually required for anterior column reconstruction for this type of deformity (3, 4). Extents of surgical trauma are relatively lower when spinal shortening is not required, because surgeons can avoid the folds of the dural sac, reducing the incidence of neurological complications.

Rigid deformity is a kind of locally stable kyphosis caused by vertebrae becoming wedge-shaped during the fracture healing process. These cases can be corrected by a single posterior surgery. Whether anterior reconstruction is needed depends on the kyphosis angle and the selection of specific osteotomy techniques. Rigid deformity kyphosis correction requires shortening of the spinal column, which can lead to dural sac folds, increasing the risk of neurological complications.

In this paper, the authors introduce a new surgical technique—reducible deformity—for old thoracic AO type B2.3 fractures. To the authors' knowledge, this is the first report describing this technique for reducible deformities. This operation can be completed through a simple posterior approach, and hemivertebral resection and reconstruction technology can restore the height of the spinal anterior column, correct kyphosis, and achieve effective fusion of the anterior column, avoiding common complications such as subsidence of the interbody fusion apparatus.

## Case presentation

**Figure 1:**
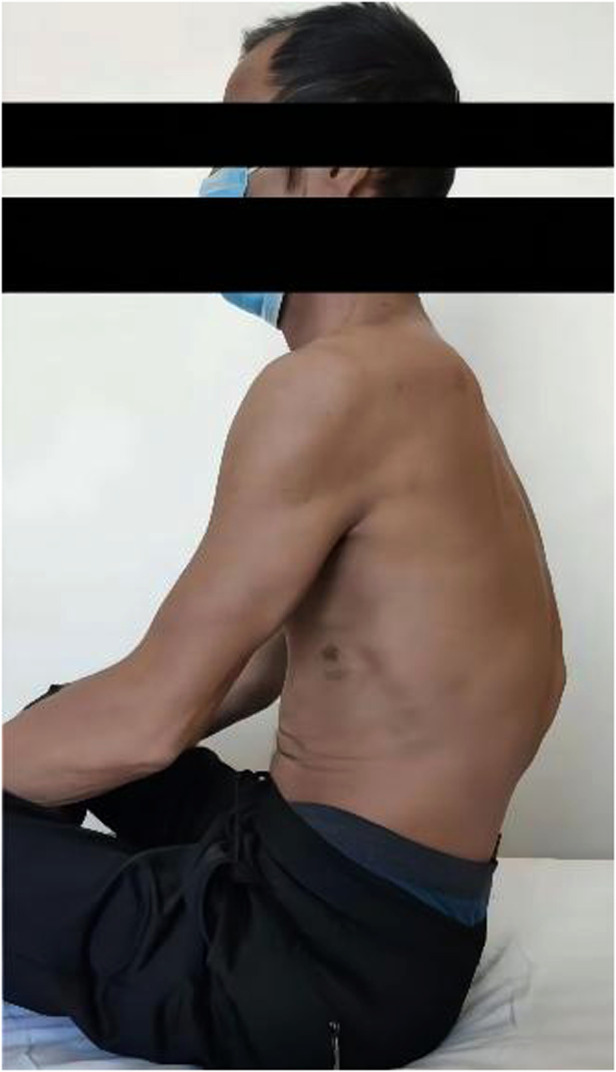
Pre-operative external observation.

**Figure 2:**
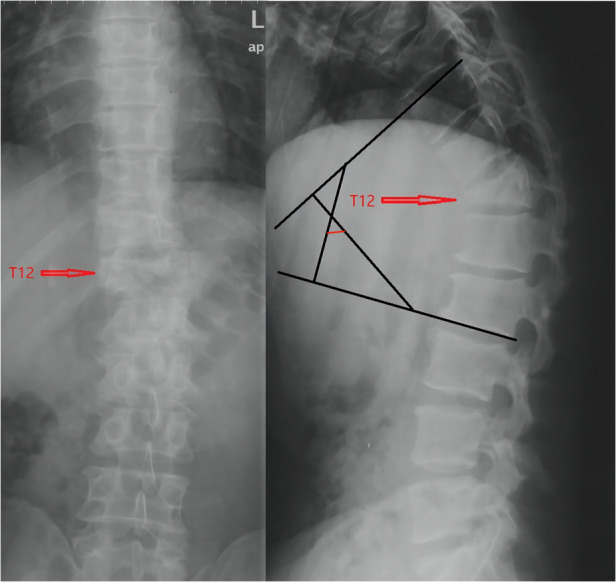
Anteroposterior and lateral radiographs of the thoracolumbar segment; Thoracolumbar cobb angle is 57°.

**Figure 3:**
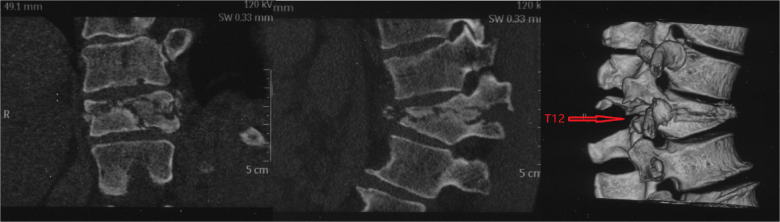
Three-dimensional CT.

**Figure 4:**
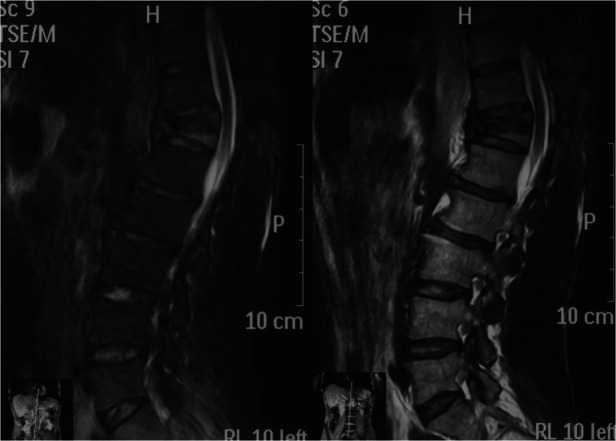
Magnetic resonance imaging.

In June 2020, a 41-year-old man was admitted to the Second Affiliated Hospital of Harbin Medical University. More than two months prior, the patient had been injured by heavy objects. The patient developed chest and back pain and limited movement, accompanied by multiple injuries including in the right knee and right ankle. He received surgical treatment for knee and ankle joint fractures, and conservative treatment for thoracic vertebra and left clavicle fractures. Following the trauma, his chest and back pain were not completely relieved, and he gradually developed kyphosis (Figure 1). Neurological examination showed hypoesthesia of the skin on the lateral side of the right knee and right ankle and no other apparent neurological injuries. Anteroposterior and lateral radiographs of the thoracolumbar segment (Figure 2) showed that the T12 vertebrae had become wedge-shaped and the patient had developed severe kyphosis (thoracolumbar cobb angle is 57°). Three-dimensional CT (Figure 3) showed that the anterior edge of the T12 vertebral body was compressed to ¾ of the normal range, the anterior and left hemivertebra showed a severely comminuted fracture, the right hemivertebra was transversely split, and the bilateral pedicle and accessory structures had flexion-distraction injuries (FDI). Magnetic resonance imaging (Figure 4) showed fractures of T12, wedge-shaped vertebrae, and no apparent spinal cord compression. He was diagnosed with an old AO type B2.3 thoracic fracture based on imaging findings of posterior disruption of the osseous tissue, with vertebral body compression. He had an LSC score of 8 based on the following: 30%–60% comminution (2 points), fragments of at least 2 mm in size which were displaced >50% of the cross section of the structure (3 points), and kyphotic correction of ≥10° (3 points) (2). His chest and back VAS score was 6, and an ODI score could not be calculated accurately because the affected limb was accompanied by multiple unhealed fractures of the right lower limb. The patient had chest and back pain that did not respond to conservative treatment, unhealed local fractures and an unstable spine. Based on the above indicators, surgery was planned.

The patient was placed in a prone position after general anesthesia. A longitudinal incision was made with T10-L2 as the center, and a total of eight suitable pedicle screws were inserted into the bilateral pedicles of T10, T11, L1, and L2. Approximately 3 cm of the left rib connected to T12, the left transverse process of T12, the lamina, the facet joint, and the left half vertebra of T12 were removed with an ultrasonic bone knife. The left side of the T11/12 and T12/L1 intervertebral discs were removed *via* repeated curetting to expose the bony endplate. A bone chisel was inserted into the broken end of the right T12 vertebral body from the left side for local release. The right pedicle screw was implanted with a titanium rod under the radian convex side, and the local kyphosis was corrected by the pull-reduction technique. Autologous granular bone was grafted between the intervertebral spaces of T11/12 and T12/L1 near the midline, and onto the fractured ends of the right vertebral body of T12. Autologous bone trimmings obtained from decompression was made into granular bone, and an appropriate titanium cage was filled and placed at the left edge of the T11-L1 gap. Titanium rods of suitable length were pre-bent to fit the physiological curvature of the spine and placed into the bilateral pedicle screw openings. Local moderate pressure was applied, and a crosslinking device was installed after locking with the top wire. C-arm fluoroscopy showed that the screws and titanium rods were well-positioned and that the length was suitable. The wound surface was repeatedly washed, two rubber tubes were placed beside the spinous process for drainage, and hemostasis was performed. Layered suturing was used to close the incision. Open reduction and internal fixation of the left clavicle fracture were performed in the supine position, and the patient was returned to the ward. Postoperative anti-infection, analgesic, and symptomatic treatments were performed as necessary, and one week after surgery, the patient was able to move out of their bed while supported.

**Figure 5:**
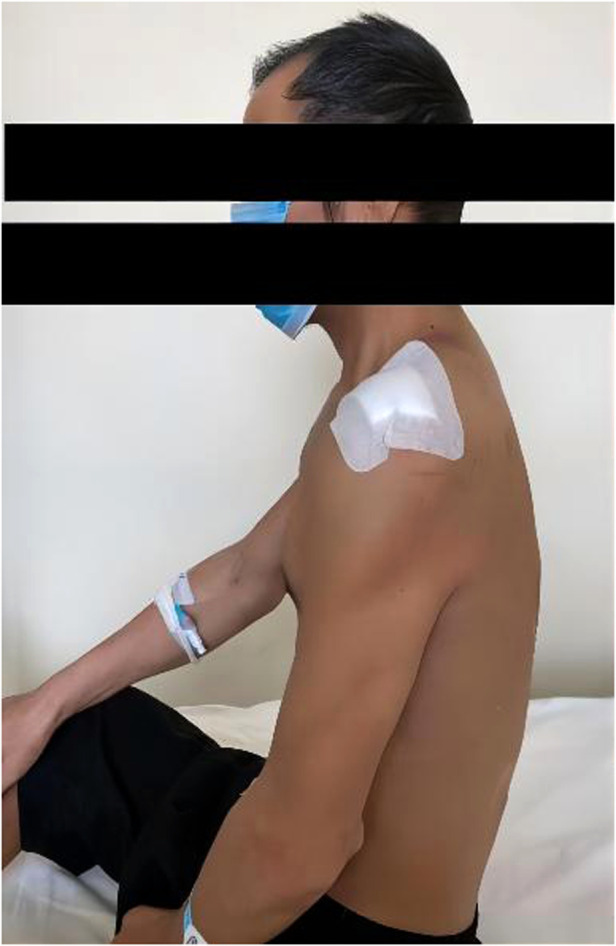
Post-operative external observation.

**Figure 6:**
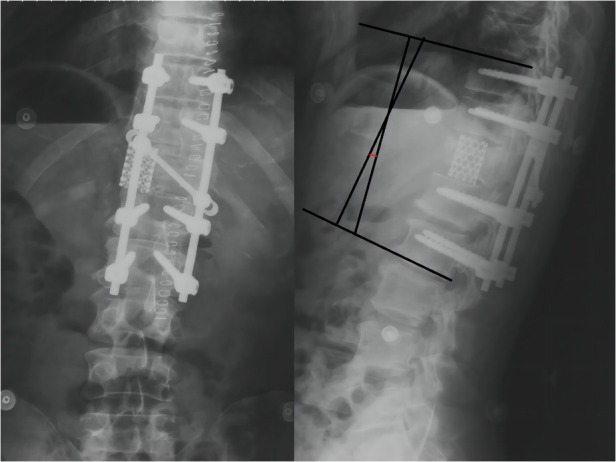
Anteroposterior and lateral radiographs of the thoracolumbar segment; Thoracolumbar cobb angle is 11° (One week after surgery).

**Figure 7:**
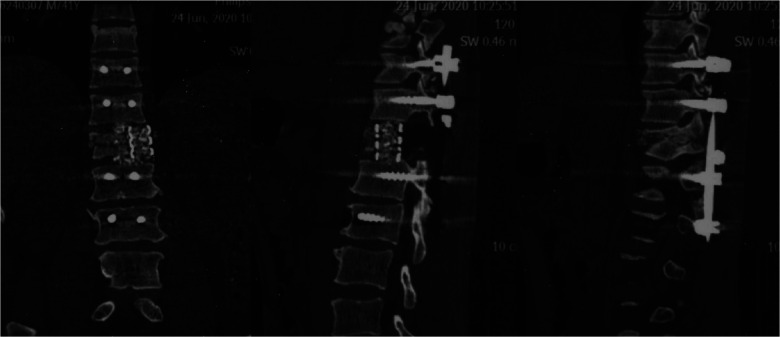
Sagittal and coronal CT of thoracic vertebra (One week after surgery).

**Figure 8:**
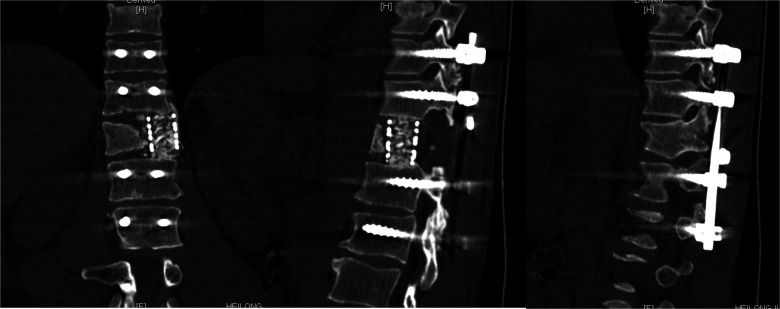
Sagittal and coronal CT of thoracic vertebra (Nine months after surgery).

**Figure 9:**
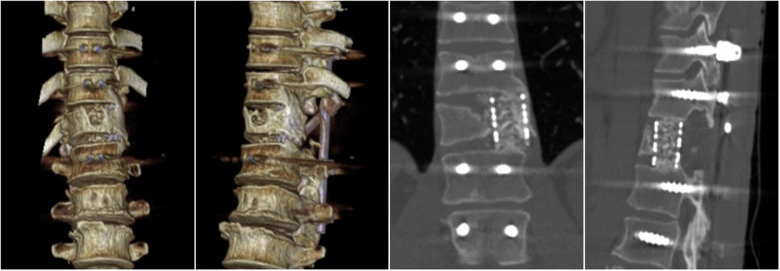
Thoracolumbar 3D CT (Eighteen months after surgery).

One week after the operation, external observation images (Figure 5) were taken. Re-examination of thoracolumbar x-rays (Figure 6) and thoracolumbar 3D CT (Figure 7) showed that the physiological curvature of thoracolumbar was restored(thoracolumbar cobb angle is 11°), the fracture end of the right pedicle and posterior vertebral body of T12 was closed, and the left half of the vertebral body was well reconstructed. Nine months after surgery, the patient had no apparent thoracolumbar discomfort and had recovered well. A review of his thoracolumbar 3D CT (Figure 8) showed no significant changes in the physiological curvature of the thoracic vertebrae, good titanium cage positioning, continuous callus formation between adjacent vertebrae, and bony fusion of the fractured ends of the residual vertebrae. Eighteen months after the operation, 3D CT of the thoracolumbar segment was reviewed (Figure 9) and showed that the titanium cage was surrounded by the callus and had fused with the adjacent vertebrae.

## Discussion and conclusions

In the AO classification system, FDIs are defined as b-type damage. FDI are usually caused by frontal shear of the frontal column or the frontal column rotation axis, and are characterized by posterior and middle column damage or tripillar damage (5). This type of injury has poor stability, and incorrect or delayed treatment may cause post-traumatic fracture nonunion and kyphosis, which is not uncommon in clinical practice. Many patients may develop back pain and neurological dysfunction, requiring surgical treatment (6). Our procedural objective was to restore the normal physiological curvature of the spine and sagittal and coronal balance of the vertebral body, prevent further malformation development, and relieve symptoms of spinal nerve compression. Pakrer (7) believed that success of malformation correction procedures largely depends on the choice of surgical approach. The surgical approach can be a simple anterior approach, simple posterior approach, or a combined anterior and posterior approach. LSC has been effectively used in determining the choice of surgical approach. Patients with an LSC score <6 points should use a posterior approach, and patients with an LSC score >6 points should use an anterior approach. Adherence to these guidelines has improved the success of surgical reduction and fixation, decreasing rates of recurrence and/or fixation failure of kyphosis (7–9). However, the surgical risk and technical difficulty of the anterior approach are higher than those of the posterior approach, so the approach and surgical method to be adopted depends on the surgeon's proficiency in different surgical techniques and the specific injury context (5).

Osteotomy and orthopedic technology can be divided into six levels of difficulty and risk according to the volume of osteotomy needed and the kyphosis angle. The representative procedures for each of the six difficulty levels, in order of increasing difficulty, are the Smith-Petersen osteotomy (SPO), Ponte osteotomy, pedicle subtraction osteotomy (PSO), Bone-Dis-Bone osteotomy (BDBO), vertebral column resection (VCR) and multiple adjacent vertebrae and discs resection (VCRs) (10). Different osteotomy methods can be used for various diseases. For diseases with angular kyphosis, a higher-level procedure is generally required, which often require significant shortening of the posterior column at the surgical site and subsequent folding of the dural sac. These procedures are commonly associated with neurological complications such as spinal cord injury, spinal spondylolisthesis, and postoperative nail and rod breakage (11, 12). In recent years, Ding (13–15) and other scholars put forward unilateral posterior vertebral column resection (UPVCR) bone cutting technology; this technique excises the ipsilateral and most of the contralateral vertebrae obliquely through a unilateral approach, with approximately 330° decompression, and can be applied to angular kyphosis, Kummell's disease in elderly patients, and kyphosis correction treatment. Compared with posterior vertebral column resection (PVCR), UPVCR has advantages such as shortened operation times, reduced blood loss, and reduced incidence of nerve root injury, while achieving satisfactory correction of sagittal malformations, improvement of function, and pain relief.

In this case, the patient had an old AO type B2.3-old thoracic fracture, with a posterior osseous structure FDI combined with a type A vertebral fracture. The patient did not receive immediate treatment after his trauma, resulting in nonunion of the fracture and thoracolumbar kyphosis and bone absorption imaging of the left vertebral body. In this paper, the author applied hemivertebral resection and reconstruction techniques in the orthopedic treatment of kyphosis caused by an old AO type B2.3 thoracic fracture. The scope of resection of the vertebral body with this technique was smaller than that of UPVCR. Intraoperative resection of the type A damaged vertebral body was carried out through titanium cage reconstruction, and a bone pick was used to pry open the contralateral fracture end. The residual posterior fracture end was closed and reduced by pull-reduction and compression technology, while the anterior compression site was further opened. Autologous bone trimmings were grafted onto the intervertebral space and fracture space to achieve the best reduction and fusion. The author had the following recommendations regarding this technique: 1. The portion of the vertebral body that contained a type A injury was selected as the resection side; 2. The area of resection should not exceed the midline of the vertebral body; 3. The titanium cage was implanted at the edge of the vertebral body; and 4. The contralateral joint structure was fully retained. The above comments are based on the following theories: 1. In type B2.3 fracture combined with type A injury, most of the injured side is associated with a damaged cartilage endplate, making it unsuitable for structure retention due to poor local stability; 2. The titanium cage is placed at the edge of the vertebral body to retain as much original bone as possible, as this part of the bone has a strong compression resistance ability and can prevent subsidence of the titanium cage; 3. Retaining the contralateral facet structure can further increase intervertebral stability.

Hemivertebral resection and reconstruction is an orthopedic technique for kyphosis that can fully preserve intervertebral height and generate highly efficient fusion. It can be used in the orthopedic treatment of rigid kyphosis and Kummell's disease in elderly patients, as well as in anterior column reconstruction to alleviate kyphosis caused by a traumatic spinal fracture. This operation can be completed through the posterior approach and requires a smaller extent of surgical incision while offering strong local stability, less blood loss, and fewer neurological complications. Based on these advantages, this technique is worthy of further usage in the clinic for treatment of thoracolumbar spine fractures.

## Data Availability

The original contributions presented in the study are included in the article/Suplementary Material, further inquiries can be directed to the corresponding author/s.
